# ^13^N-Ammonia PET-CT for Evaluating Response to Antiangiogenic Therapy and Prognosis in Patients with Advanced Hepatocellular Carcinoma: A Pilot Study

**DOI:** 10.3390/cancers17040656

**Published:** 2025-02-15

**Authors:** Valentina Scolozzi, Alberto Nicoletti, Amedeo Capotosti, Francesca Romana Ponziani, Silvia Taralli, Enza Genco, Lucia Leccisotti, Roberto Moretti, Luca Indovina, Maurizio Pompili, Maria Lucia Calcagni

**Affiliations:** 1 Unità di Medicina Nucleare, Dipartimento di Diagnostica per Immagini e Radioterapia Oncologica, Fondazione Policlinico Universitario A. Gemelli IRCCS, 00168 Rome, Italy; 2 Liver Unit, CEMAD-Centro Malattie dell’Apparato Digerente, Medicina Interna e Gastroenterologia, Fondazione Policlinico Universitario A. Gemelli IRCCS, 00168 Rome, Italy; 3 Dipartimento di Diagnostica per Immagini e Radioterapia Oncologica, Fondazione Policlinico Universitario A. Gemelli IRCCS, 00168 Rome, Italy; 4 Dipartimento di Medicina e Chirurgia Traslazionale, Università Cattolica del Sacro Cuore, 00168 Rome, Italy; 5 Radiologia Addomino-Pelvica, Dipartimento di Diagnostica per Immagini e Radioterapia Oncologica, Fondazione Policlinico Universitario A. Gemelli IRCCS, 00168 Rome, Italy; 6 Istituto di Medicina Nucleare, Dipartimento Universitario di Scienze Radiologiche ed Ematologiche, Università Cattolica del Sacro Cuore, 00168 Rome, Italy

**Keywords:** ^13^N-ammonia PET-CT, hepatocellular carcinoma, antiangiogenic therapy, response evaluation, prognosis

## Abstract

Purpose: To prospectively investigate dynamic ^13^N-ammonia PET-CT for evaluating early treatment response and predicting prognosis in advanced hepatocellular carcinoma (HCC) patients who have undergone antiangiogenic therapy. Methods: Dynamic ^13^N-ammonia PET-CT was performed in 23 advanced HCC patients before antiangiogenic therapy (baseline) and in 18/23 patients after 8-10 weeks of treatment (post-therapy). At kinetic PET-CT analysis, mean, maximum, and peak values of *K_1_* (mL/cm^3^/min) and *k_2_* (min^−1^) were estimated in HCC lesions and non-neoplastic liver using cardiologic ^13^N-ammonia PET-CT in 15 patients without any liver diseases as normal controls. Outcome endpoints were treatment response after 8–10 weeks assessed by contrast-enhanced CT, progression-free survival (PFS), and overall survival (OS). Results: At both baseline and post-therapy PET-CT, all kinetic PET parameters were significantly higher (*p* < 0.05) in HCC lesions than in non-neoplastic and healthy liver of HCC patients and controls. According to mRECIST criteria, 13/18 patients (72.2%) were responders (1 CR, 1 PR, and 11 SD), and 5/18 patients (27.8%) were non-responders (PD), with no significant differences in baseline and post-therapy PET parameters between the two groups. At follow-up (median: 14.2 months), 15/18 patients (83.3%) experienced radiological progression, and 14/18 (77.8%) died (7/14 within 12 months from treatment). The nine earlier-progression patients (within 6 months from treatment) showed significantly lower baseline *K_1mean_* in HCC lesions than all nine patients with later or no-progression (*p* = 0.03). Patients still alive 12 months after treatment (n = 11) showed significantly lower post-therapy *K_1mean_* (*p* = 0.05), *K_1max_* (*p* = 0.05), and *K_1peak_* (*p* = 0.03) in non-neoplastic liver than patients with shorter OS (n = 7). Conclusions: In advanced HCC patients treated with antiangiogenic agents, kinetic parameters from baseline and post-therapy ^13^N-ammonia PET-CT may predict early disease progression and survival. PET-CT seems not able to discriminate responders and non-responders after 8-10 weeks of treatment, suggesting the need for future and larger studies after a longer treatment period.

## Simple Summary

The aim of this prospective study was to investigate the role of kinetic parameters derived from dynamic ^13^N-ammonia PET-CT for evaluating early treatment response and predicting prognosis in patients with advanced hepatocellular carcinoma (HCC) treated with antiangiogenic therapy. Patients with earlier radiological progression showed lower baseline *K_1mean_* in HCC lesions. Patients with longer overall survival showed significantly lower post-therapy *K_1__mean_*, *K_1__max_*, and *K_1__peak_* values in non-neoplastic liver parenchyma. However, kinetic parameters could not distinguish between responders and non-responders after 8–10 weeks of treatment. This study suggests a potential role of ^13^N-ammonia PET-CT in selecting advanced HCC patients that will benefit from antiangiogenic therapy.

## 1. Introduction

Hepatocellular carcinoma (HCC) is the most common primary liver tumor in Western countries, generally developing on the cirrhosis background [1]. In patients with advanced HCC (30–35% of patients at diagnosis) according to the Barcelona Clinic Liver Cancer staging system (BCLC C), for vascular invasion or extrahepatic disease with preserved liver function and normal/intermediate performance status (PS), the combination of bevacizumab (an inhibitor of the vascular endothelial growth factor receptor) and atezolizumab (a PD-L1/PD-1 immune checkpoint inhibitor) has been recently introduced as first-line therapy [2,3]. Nevertheless, antiangiogenic therapy with sorafenib (a multi-tirosinkinase inhibitor reducing tumor proliferation and angiogenesis) or lenvatinib (a multikinase inhibitor targeting simultaneously some biomolecular pathways) remains the treatment of choice in case of contraindication to immune checkpoint inhibitors or progression after the bevacizumab/atezolizumab combination [4,5,6]; antiangiogenic agents can also be considered in intermediate HCC patients (BCLC B) not responding to locoregional treatments (e.g., transarterial chemoembolization or radioembolization) [4]. The development of biomarkers that can accurately predict the efficacy of antiangiogenic therapy in terms of both tumor response and prognosis is crucial due to its high cost and prevalence of adverse effects and limited efficacy in some subpopulations. Since the reduction in tumor vascularization is the primary effect of antiangiogenic therapy, assessing perfusion changes should help early response evaluation. In clinical practice, antiangiogenic therapy efficacy is generally evaluated by contrast-enhanced computed tomography (c.e. CT) two months after the treatment start, according to mRECIST criteria that are based on dimensional changes in viable tumor size (i.e., the portion of target lesions with residual arterial enhancement) [6,7]. However, antiangiogenic agents can determine an irregular decline of tumor vascularization not resulting in necrotic tissue, limiting radiological response evaluation, especially in irregularly shaped HCC lesions. In this challenging context, the need emerges for additional imaging tools able to early detect tumor perfusion changes and also to predict prognosis. In the functional imaging field, ^13^N-ammonia is a perfusion Positron Emission Tomography (PET) tracer mainly metabolized in the liver [8,9,10]. In the oncological setting, ^13^N-ammonia PET was applied for assessing tumor perfusion, proving that several neoplasms show increased ^13^N-ammonia uptake (depending on tumor vascularization and tracer extraction efficacy), also suggesting a potential role for assessing response to treatments acting on tumor perfusion, especially if the baseline tracer uptake is sufficiently high [11,12,13]. Interestingly, HCC is a hypervascular tumor, and several enzymes involved in nitrogen metabolism (e.g., carbamyl-phosphate-synthetase and glutamine-synthetase) are overexpressed in HCC cells [14,15]. These characteristics make ^13^N-ammonia the ideal tracer for HCC evaluation, also considering its short half-life (10 min), implying good dosimetry. Conversely, its short half-life allows the use of ^13^N-ammonia only in PET centers equipped with a cyclotron, since it needs on-site tracer production. This practical aspect reasonably limits the widespread use of this tracer and, consequently, the amount of available related literature. The few and “historical” studies (before the clinical introduction of antiangiogenic drugs) that analyzed the role of ^13^N-ammonia in HCC patients demonstrated a relationship between tumor blood flow and tracer uptake [16,17].

The aims of this prospective study were to investigate the role of kinetic parameters derived from dynamic ^13^N-ammonia PET-CT for evaluating early response to antiangiogenic therapy and for predicting prognosis in advanced HCC patients.

## 2. Materials and Methods

### 2.1. Patients

The prospective study protocol has been approved by the Ethics Committee of the Fondazione Policlinico Universitario “Agostino Gemelli” IRCCS of Rome, Italy (28603/19, ID 2670). From July 2019 to December 2021, 23 consecutive advanced HCC patients with preserved (Child-Pugh A) or moderately impaired (Child-Pugh B) liver function and Eastern Cooperative Oncology Group (ECOG) PS ≤ 2 were candidates to start antiangiogenic therapy at the local Liver Diseases Outpatient Clinic. Exclusion criteria were contraindication to antiangiogenic therapy, history of other malignancies, contraindication to c.e. CT, and females of childbearing age. Written informed consent was obtained from all enrolled patients. ^13^N-ammonia PET-CT and chest and abdomen c.e. CT were performed before starting antiangiogenic therapy (within 15 days) and after 8–10 weeks of treatment. According to institutional multidisciplinary liver tumor board re-evaluation, antiangiogenic therapy was continued in patients with stable or responding disease, stable liver function, and adequate PS (ECOG < 2). Clinical, laboratory, and imaging data were collected at recruitment and during follow-up; radiologic progression and death were recorded as outcomes until September 2023.

### 2.2. ^13^N-Ammonia PET-CT

All ^13^N-ammonia PET-CT scans were performed at the local PET-CT Center, using the Biograph mCT (Siemens Healthineers, Chicago, IL, USA) PET-CT scanner system, with the upper abdomen in the field of view. A 3D dynamic list-mode acquisition lasting 20 min started simultaneously with the i.v. administration of ^13^N-ammonia (370 MBq). Images were reconstructed with the following framing: 9 frames of 5 s each, 1 frame of 30 s, 1 frame of 45 s, 6 frames of 3 min each. PET data were corrected for decay, scatter, random events, dead time, and attenuation and reconstructed with the Ordered Subset Expectation Maximization (OSEM) algorithm, including Time-of-Flight (ToF) and Point Spread Function (PSF) modeling with 21 subsets and 2 iterations, with a 256 × 256 matrix size (3.18 × 3.18 × 5 mm) and applying a 2 mm post-reconstruction Gaussian filter. A static image was also reconstructed. Before PET acquisition, a low-dose CT scan was performed (tube current 50 mAs, voltage 120 kV, pitch 0.9, exposure time 0.5 s) for attenuation correction and morphological information. Kinetic analysis of dynamic PET images was performed using Volumes of Interest (VOIs). For each patient, HCC lesions were manually contoured on c.e. CT images, employing MIM software (v.7.3.4) to achieve precise lesions’ delineation and forming the baseline for subsequent fusion and contouring tasks. Exploiting both rigid and deformable registration capabilities of the MIM software, c.e. CT images were fused with dynamic PET-CT series, facilitating the automatic contour transfer to the dynamic PET-CT series; manual editing on the PET-CT series ensured accuracy and precision of contours. In patients with multiple HCC lesions, the largest one was chosen (HCC VOI). Moreover, the uptake of non-neoplastic liver parenchyma was evaluated by a spherical VOI of 1 cm^3^ placed on normal-appearing liver parenchyma on c.e. CT images and subsequently transferred to PET images (liver VOI). A spherical VOI with the same volume was placed in liver parenchyma to extract the kinetic parameters in a local population of 15 patients who underwent ^13^N-ammonia PET-CT for cardiologic indications and without known liver diseases, used as normal controls. Finally, to construct the Image-Derived Input Function (IDIF), a VOI was manually delineated on the descending aorta, placing three consecutive Regions of Interest (ROI). An evaluation between 2-tissue and 1-tissue compartment models was performed with PKIN to assess the best fit with patients’ data. Kinetic analysis was then conducted using the PXMOD tool, choosing the 1-tissue compartment Alpert model (i.e., a slightly modified version of the classical implementation of Alpert’s time-weighted integral approach for brain perfusion) [18,19] because it was the best fit; this model operates on the principle of a single exchange between blood and tissue compartments and provides the kinetic PET parameters *K_1_* (mL/cm^3^/min) and *k_2_* (min^−1^), respectively, representing the ^13^N-ammonia blood-to-tissue and tissue-to-blood transport (liver as tissue of interest). The parametric maps for both *K_1_* and *k_2_* (representing the spatial distribution of the kinetic parameters) were automatically generated, illustrating the quantitative behavior of the tracer over time; HCC and liver VOIs were loaded onto these maps, and the mean, maximum, and peak values of *K_1_* and *k_2_* from each VOI were extracted.

### 2.3. Antiangiogenic Therapy

All patients started on antiangiogenic therapy with either sorafenib (start dosage: 400 mg bid) or lenvatinib (start dosage: 8–12 mg qd, according to body weight < or >60 Kg), with dosage reduction in case of adverse effects or liver function test abnormalities. Treatment was discontinued in case of clinical liver decompensation, deterioration of patients’ PS, or severe liver function test alterations.

### 2.4. Statistical Analysis

Given the absence of previous similar studies on ^13^N-ammonia PET-CT in patients with advanced HCC undergoing antiangiogenic treatment (i.e., the pilot nature of this study), there was no need for a formal calculation of a minimum sample size. However, consistent with proper statistical rules [20], we planned to enroll at least 18 patients, considering the mean annual accesses of advanced HCC patients eligible for antiangiogenic therapy to the local Liver Diseases Outpatient Clinic and a 25% treatment discontinuation before the first imaging follow-up (8 weeks) due to adverse effects.

Response to antiangiogenic treatment were assessed using mRECIST criteria [7] on c.e. CT performed at 8-10 weeks from the start of treatment; patients’ response was classified into four categories: complete response (CR), partial response (PR), stable disease (SD) or progressive disease (PD), then grouping CR, PR, and SD into the “responders” category, and PD into the “non-responders” one. Patients were followed up until death or until September 2023 in still-living patients. Progression-free survival (PFS) and overall survival (OS) were, respectively, defined as the time (months) from the start of treatment to radiologic progression (at c.e. CT or magnetic resonance) or to death during follow-up. For patients who had not experienced recurrence or death at the time of the last follow-up, PFS and OS were censored at the last follow-up date. Patients’ characteristics were reported according to descriptive data analysis techniques. Quantitative variables were described as median and interquartile range (IQR) or mean ± standard deviation (SD), using the Smirnov–Kolmogorov test to assess normal distribution. Qualitative variables were defined as absolute frequency or percentage. According to the Smirnov–Kolmogorov test result, the association between dependent (response to treatment, PFS, OS) and independent variables (kinetic PET parameters) was tested using univariate analysis by Wilcoxon and Mann–Whitney tests; comparison between binary variables was tested using Fisher’s exact test. PET parameter performance was also assessed by receiver operating characteristic (ROC) curve analysis, using PFS at 6 months and OS at 12 months as a reference; the decision cut-off was considered optimal when the product of paired values for sensitivity and specificity reached its maximum. A *p*-value < 0.05 was considered statistically significant. Data analysis was performed using MedCalc software (version 11.6; Broekstraat, Mariakerke, Belgium).

## 3. Results

### 3.1. Patient Characteristics

**Figure 1:**
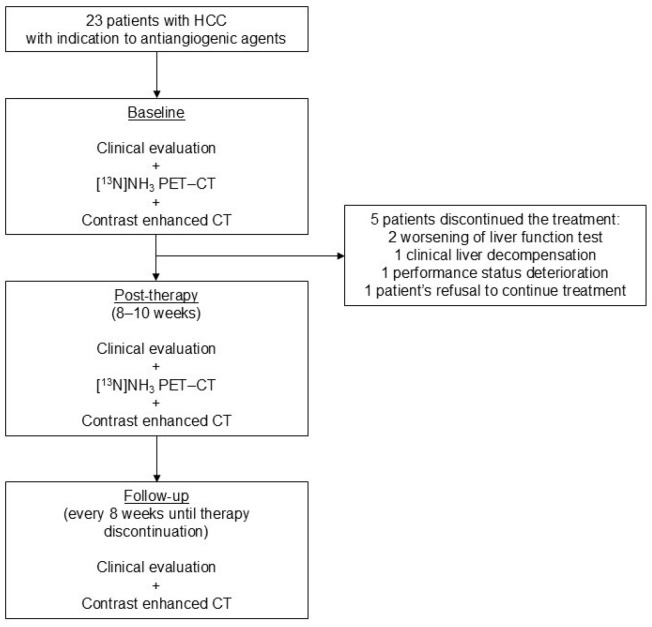
Study design and population.

The clinical characteristics of the study population (n = 23) are summarized in Table 1 and Figure 1. All patients were cirrhotic and underwent a baseline ^13^N-ammonia PET-CT scan. Thirteen (56.5%) patients were started on sorafenib and ten (43.5%) on lenvatinib. In one patient (7.7%) on sorafenib treatment and four patients (40%) on lenvatinib treatment, the dosage was reduced during treatment. Treatment was stopped before 8 weeks in 5/23 patients (21.7%) because of worsening liver function tests (n = 2), clinical liver decompensation (n = 1), PS deterioration (n = 1), or patient’s refusal to continue treatment (n = 1). Therefore, post-therapy ^13^N-ammonia PET-CT was performed in 18/23 patients (78.3%).

### 3.2. Kinetic PET Parameters in HCC Lesions and Non-Neoplastic Liver Parenchyma

**Figure 2:**
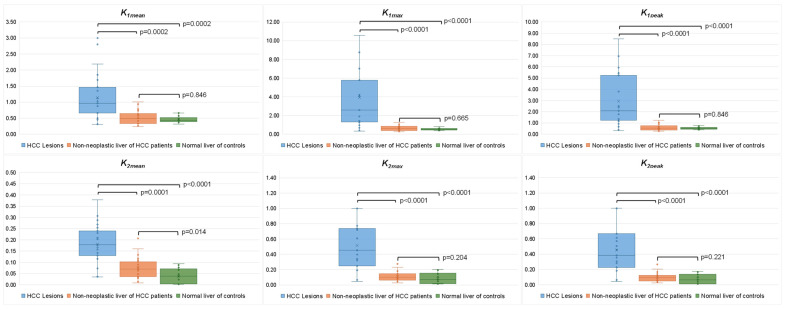
Comparison between baseline kinetic PET parameters of HCC lesions (n = 23), non-neoplastic liver of HCC patients (n = 23), and normal liver parenchyma of controls (n = 15).

The best fit of the 2-tissue and 1-tissue compartment models to the patient data showed median percentage SE for *k_3mean_* and *k_4mean_* of 2.31 × 10^24^ (IQR 2.66 × 10^6^–1.94 × 10^30^) and 4.00 × 10^3^ (IQR 8.26 × 10^2^–7.36 × 10^5^), respectively, while the median percentage SE for *k_1mean_* and *k_2mean_* were 5.02 (IQR 3.74–7.23) and 7.75 (IQR 5.71–12.1), respectively. Due to the large percentage SE for *k_3mean_* and *k_4mean_*, the 1-tissue model was selected to perform the following pixelwise parametric compartmental model calculation. At baseline ^13^N-ammonia PET-CT (n = 23), all kinetic PET parameters were significantly higher in HCC lesions compared to non-neoplastic liver of HCC patients and liver parenchyma of normal controls. A significant difference in *k_2mean_* values between non-neoplastic liver and the liver of normal controls was found (0.069 (IQR 0.036–0.101) vs. 0.038 (IQR 0.003–0.070), *p* = 0.014). However, no significant difference was observed in the other PET parameters (Figure 2).

**Figure 3:**
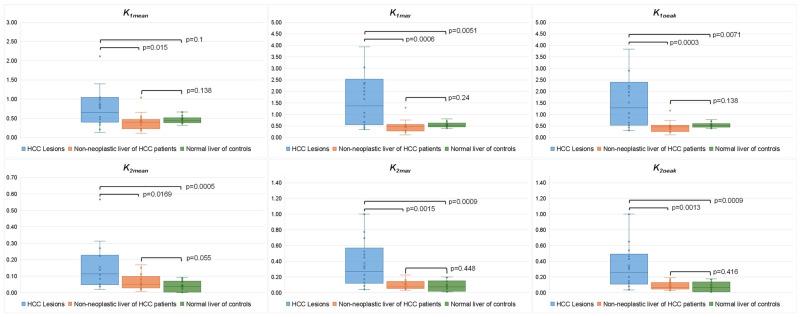
Comparison between post-therapy kinetic PET parameters of HCC lesions (n = 18), non-neoplastic liver of HCC patients (n = 18), and normal liver parenchyma of controls (n = 15).

At post-therapy ^13^N-ammonia PET-CT (n = 18), all PET parameters remained significantly higher in HCC lesions compared to non-neoplastic liver and controls. No significant difference was found in PET parameters between the non-neoplastic liver of HCC patients and the liver parenchyma of control subjects, with only a trend for higher *k_2mean_* values in the HCC group (0.050 (IQR 0.028–0.098) vs. 0.038 (IQR 0.003–0.070), *p* = 0.055) (Figure 3).

**Figure 4:**
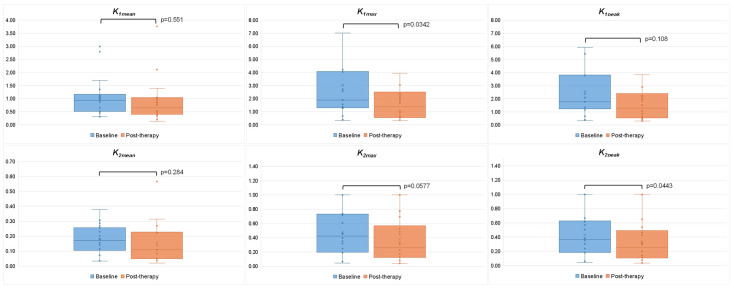
Comparison between baseline and post-therapy kinetic PET parameters in HCC lesions (n = 18).

Comparing baseline and post-therapy PET-CT parameters in HCC lesions, *K_1max_* (1.901 (IQR 1.308–4.047) vs. 1.370 (IQR 0.554–2.335), *p* = 0.034) and *k_2peak_* (0.369 (IQR 0.185–0.615) vs. 0.259 (IQR 0.118–0.478), *p* = 0.034) were significantly reduced at post-therapy compared to baseline, with also a trend towards statistical significance observed for *k_2max_* values (0.425 (IQR 0.198–0.731) vs. 0.270 (IQR 0.129–0.731), *p* = 0.0557) (Figure 4). No significant difference was found in all kinetic PET parameters of non-neoplastic liver between baseline and post-therapy PET.

### 3.3. Kinetic PET Parameters and Clinical Outcomes

Considering the 18 patients who completed at least 8–10 weeks of treatment and underwent post-treatment PET, 13/18 patients (72.2%) were classified as responders according to mRECIST criteria (1 CR, 1 PR, and 11 SD), and treatment was continued, with one patient transplanted after CR; 5/18 patients (27.8%) were classified as non-responders (all PD), and the treatment was discontinued. No significant difference was found in baseline and post-therapy dynamic PET parameters, or in their percentage variations, between responders and non-responders. When comparing kinetic parameters in the responder patients (n = 13), significant reductions in *K_2max_* and *K_2peak_* values were observed between baseline and post-therapy PET (*K_2max_* (0.397 (IQR 0.170–0.733) vs. 0.228 (IQR 0.096–0.568), *p* = 0.014) and *K_2peak_* (0.354 (IQR 0.156–0.632) vs. 0.201 (IQR 0.081–0.493), *p* = 0.02).

**Figure 5:**
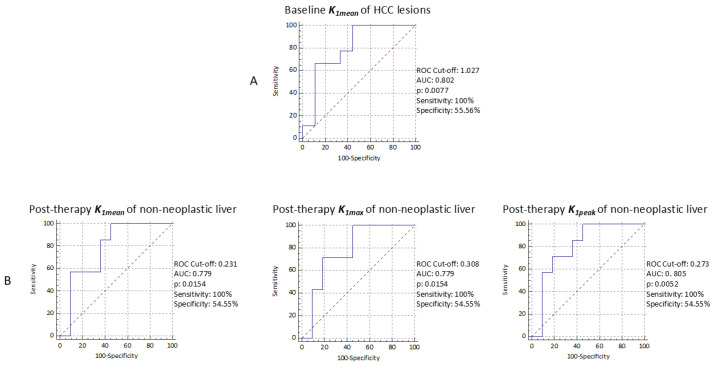
Diagnostic performance of kinetic PET parameters assessed by additional ROC curve analysis using progression-free survival at 6 months (**A**) and overall survival at 1 year (**B**) as a reference.

**Figure 6:**
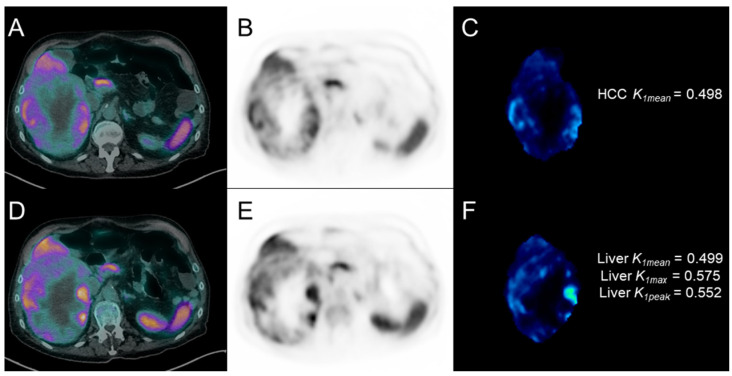
^13^N-ammonia PET images of patient #16, with a wide HCC lesion of the right liver lobe, at baseline ((**A**): transaxial PET/CT; (**B**): transaxial PET; (**C**): *K_1_* parametric map of HCC lesion) and at post-treatment ((**D**): transaxial PET/CT; (**E**): transaxial PET; (**F**): *K_1_* parametric map of non-neoplastic liver parenchyma).

**Figure 7:**
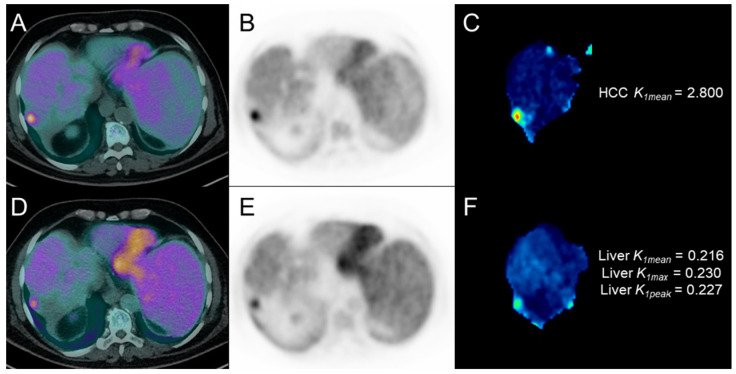
^13^N-ammonia PET images of patient #12, with an HCC lesion of the 7th liver segment, at baseline ((**A**): transaxial PET/CT; (**B**): transaxial PET; (**C**): *K_1_* parametric map of the HCC lesion) and at post-treatment ((**D**): transaxial PET/CT; (**E**): transaxial PET; (**F**): *K_1_* parametric map of non-neoplastic liver parenchyma).

During follow-up (median: 14.2 months, range: 2.7–34.3), 15/18 patients (83.3%) experienced radiological progression, nine of whom within 6 months after the start of treatment. Those nine patients with early radiological progression (within 6 months) showed significantly lower *K_1mean_* values in HCC lesions at baseline ^13^N-ammonia PET than the remaining nine patients with no-progression or late-progression (0.658 (IQR 0.487–0.973) vs. 1.117 (IQR 0.899–1.969), *p* = 0.03). Results of the ROC analysis are reported in Figure 5 (section A). No significant difference was found in all the other baseline and post-therapy PET parameters (and in their percentage variations). During follow-up, 9/18 patients (50%) presented clinical decompensation and 14/18 (77.8%) deceased; 7/14 deceased within 12 months after the start of treatment. Patients still alive 12 months after the start of treatment (n = 11) showed significantly lower *K_1mean_* (0.231 (IQR 0.216–0.443) vs. 0.461 (IQR 0.394–0.533), *p* = 0.05), *K_1max_* (0.3080 (IQR 0.246–0.506) vs. 0.569 (IQR 0.475–0.600), *p* = 0.05), and *K_1peak_* (0.273 (IQR 0.233–0.489) vs. 0.529 (IQR 0.456–0.572), *p* = 0.03) values in non-neoplastic liver parenchyma at post-therapy ^13^N-ammonia PET compared with patients with shorter survival (n = 7). Results of the ROC analysis are reported in Figure 5 (section B). No significant difference was found in all baseline and other post-therapy PET parameters in non-neoplastic liver parenchyma. Figure 6 and Figure 7 show representative cases of one patient with early disease progression and death (PFS: 5.0 months; OS: 7.5 months) and one patient with late disease progression and alive at last follow-up (PFS: 12.2 months), respectively.

## 4. Discussion

To the best of our knowledge, this is the first study that evaluated the potential role of quantitative parameters derived from ^13^N-ammonia PET to predict treatment response and clinical outcomes in HCC patients treated with antiangiogenic therapy. Our results reflect the well-known biological features of HCC lesions, characterized by hyper-vascularization and deranged nitrogen metabolism in most cases [21,22]. Indeed, HCC lesions showed higher *K_1_* and *k_2_* values, indicative of higher vascular supply and faster ammonia wash-out than non-neoplastic liver and control subjects, both at baseline and post-therapy. In addition, we found that non-neoplastic liver showed higher *k_2_* values compared with normal subjects. This faster ammonia wash-out may be due to the impaired hepatocytes’ function of cirrhotic cells, although the vascular supply is preserved, being *K_1_* values similar to that of liver parenchyma of control subjects.

In HCC lesions, antiangiogenic agents act by blocking the neoangiogenesis mechanisms and reducing the vascular supply. In this context, response to sorafenib has been evaluated by perfusion parameters from c.e. CT imaging or contrast-enhanced ultrasound, suggesting a potential role of quantitative perfusion data for assessing treatment response and clinical outcomes [23,24]. As PET imaging, we found that HCC lesions showed lower perfusion after therapy, as expressed by a reduction of *K_1max_* values, so suggesting a greater efficacy of antiangiogenic agents in more vascularized areas within the lesions, making the tumor perfusion more homogeneous. It would be interesting to evaluate whether a longer treatment period could determine a reduction also in *K_1mean_* and *K_1peak_* values, unchanged after 8–10 weeks of treatment. Moreover, HCC lesions showed slower wash-out after therapy, as expressed by a reduction of *k_2_* values, more pronounced in the responder patients, suggesting an effect of antiangiogenic agents not only on perfusion but also on cell function. Finally, although we found a reduction in kinetic parameters in HCC after 8–10 weeks of treatment, lesions remained more vascularized than non-neoplastic liver parenchyma, suggesting the need for a longer observation period to detect a PET measurable change in the tumor vascular supply.

A thorough estimation of treatment response is crucial to identify patients who benefit from antiangiogenic therapy early, as these agents are characterized by significant adverse effects and high costs. The identification of early non-responders could avoid ineffective and potentially harmful therapies [25]. However, baseline or post-therapy PET parameters did not allow for the discrimination of responders from non-responder patients, probably due to the unbalanced groups’ size.

Several efforts have been made to improve the dismal prognosis of advanced HCC patients during recent years. Although combined treatment with immune checkpoint inhibitors has been shown to provide better OS and PFS than multi-tirosinkinase inhibitors alone, antiangiogenic therapy still represents an indispensable tool in these patients. In the targeted-therapy era, a tool able to predict early progression after antiangiogenic therapy may help to choose the correct treatment in patients with advanced HCC. So far, no validated biomarkers have been found [25]. In this context, our study provides potentially useful clinical findings. Indeed, the most promising result is that patients who experienced early progression (within 6 months) after antiangiogenic therapy showed lower baseline HCC *K_1_* values compared to early non-progressors. As expected, antiangiogenic agents demonstrated a greater efficacy in highly vascularized HCC lesions compared to less vascularized ones [26]. Hence, baseline ^13^N-ammonia PET could be useful to a priori select patients that will benefit from antiangiogenic therapy, avoiding adverse effects and ineffective therapies burdened by high costs in patients with expected worse clinical outcomes. The potential role of dynamic PET perfusion imaging to identify oncological patients who will benefit from antiangiogenic treatment was previously explored in different oncological populations, such as by de Langen AJ et al. [27]. The authors evaluated the role of radiolabeled water (H_2_^15^O), another PET perfusion agent, for predicting survival benefit in patients with advanced non-small cell lung cancer treated with antiangiogenic agents, within a wide imaging protocol also including ^18^F-FDG PET and c.e. MRI. In detail, the percentage change in tumor perfusion between H_2_^15^O PET imaging at baseline and 3 weeks after treatment was correlated with PFS. From H_2_^15^O PET results, the authors found that a deeper decrease in tumor perfusion was associated with longer PFS and that perfusion information seemed to allow earlier response evaluation than CT imaging, supporting the valuable role of PET perfusion imaging.

Finally, regarding our results, higher *K_1_* values of non-neoplastic liver at post-therapy PET are associated with the occurrence of death within 12 months after the start of treatment. Interestingly, the abnormal vasoconstriction in cirrhosis with portal hypertension can lead to neoangiogenesis that increases liver vascular volume in response to inflammation and fibrosis [28,29]. The increase in non-neoplastic liver perfusion (as reflected by higher *K_1_* values) might express a condition of stress, explaining a higher rate of decompensation and subsequent death in this subgroup of patients.

This study has some limitations, mainly the already mentioned small study population; this aspect may limit the statistical power of our analysis, especially when comparing responders (n = 13) vs. non-responders (n = 5), reasonably explaining the lack of significant differences in PET parameters between these two groups. Moreover, the limited number of patients allowed us to perform only a descriptive survival data analysis. However, this is the first study evaluating ^13^N-ammonia PET-CT in advanced HCC patients treated with antiangiogenic therapy, designed as a pilot study, with a sample size defined with a statistical method [20]. Secondly, we limited the PET response evaluation to the first 8-10 weeks after the start of treatment, aiming to intercept early PET measurable perfusion changes; hence, no information on perfusion changes after a longer antiangiogenic therapy period is available, and it should be explored in future studies.

## 5. Conclusions

This study suggests that, in advanced HCC patients treated with antiangiogenic agents, kinetic parameters from baseline and post-therapy ^13^N-ammonia PET-CT may help to predict early disease progression and survival; therefore, ^13^N-ammonia PET-CT may be a promising tool to select patients with advanced HCC for antiangiogenic therapy. PET parameters seem not able to discriminate responders and non-responders after 8–10 weeks of treatment; information on perfusion changes after longer antiangiogenic treatment periods should be explored in future studies and in larger populations.

## Figures and Tables

**Table 1:** Patients and tumor baseline characteristics.

Characteristics (n = 23)	Value (%)
Age (years)	68.2 ± 15.3
Male gender	21 (91.3%)
Cirrhosis	23 (100%)
Etiology	
HCV	6 (26.1%)
HBV	5 (21.7%)
Alcohol	5 (21.7%)
NAFLD	5 (21.7%)
HCV + alcohol	1 (4.3%)
Cryptogenic	1 (4.3%)
Extrahepatic spread at baseline CT	13 (56.5%)
Portal vein tumor thrombosis at baseline CT	5 (21.7%)
Child-Pugh score	
A	18 (78.2%)
B	2 (8.7%)
N/A	3 (13.1%)
BCLC	
B	6 (26.1%)
C	17 (73.9%)
ECOG performance status	
0	12 (52.2%)
1	9 (39.1%)
2	2 (8.7%)

HCV = hepatitis C virus; HBV = hepatitis B virus; NAFLD = non-alcoholic fatty liver disease; N/A = not applicable; BCLC = Barcelona Clinic for Liver Cancer; ECOG = Eastern Cooperative Oncology Group. Continuous variables are expressed as means ± SD, and categorical variables as numbers and percentages.

## Data Availability

The raw data supporting the conclusions of this article will be made available by the authors on request.
